# Functional diversity of fish in the Yuan-Red River: Role of species turnover and trait shifts

**DOI:** 10.1016/j.isci.2026.115418

**Published:** 2026-03-20

**Authors:** Xiao-Xia Huang, Ai-Ling Yang, Bin Kang, Ke-Jian He, Xiao-Han Mei, Wen-Xian Hu, Hung-Du Lin

**Affiliations:** 1 College of Water Conservancy, Yunnan Agricultural University, Kunming 650201, China; 2 Baoshan Meteorological Bureau, Baoshan 678000, China; 3 College of Fisheries, Ocean University of China, Qingdao 266003, China; 4 School of Earth Science, Yunnan University, Kunming 650091, China; 5 Kunming University of Science and Technology, Kunming 650500, China; 6 College of Fisheries, Guangdong Ocean University, Zhanjiang 524088, China

**Keywords:** environmental science, ecology

## Abstract

Understanding how environmental change and species introductions shape functional diversity is essential for conserving freshwater biodiversity. This study examines these drivers in the upper Yuan-Red River Basin over three decades (1990–2020). Using morphological traits from fish specimens, we applied generalized least squares models, hypervolume analysis, and variation partitioning to assess trait variation, functional space reorganization, and contributions of native versus non-native species. Functional space overlap between periods was only 20.6% for natives, while non-natives expanded rapidly, increasing overlap with natives from minimal to 40% by the 2020s. Locomotion traits associated with precipitation and non-native richness; feeding and habitat traits with dams, non-native richness, and water quality. Diversity indices remained stable and climatically driven, despite underlying trait restructuring. Conservation should prioritize maintaining natural flow regimes.

## Highlights

• Fish functional space in YRRB has been significantly reorganized over the past three decades
• Climate and human drivers shape functional diversity through distinct pathways
• Functional traits show divergent responses to environmental drivers
• Natural flow regimes buffer climate impacts in tropical mountain rivers

## Graphical abstract

**Figure:**
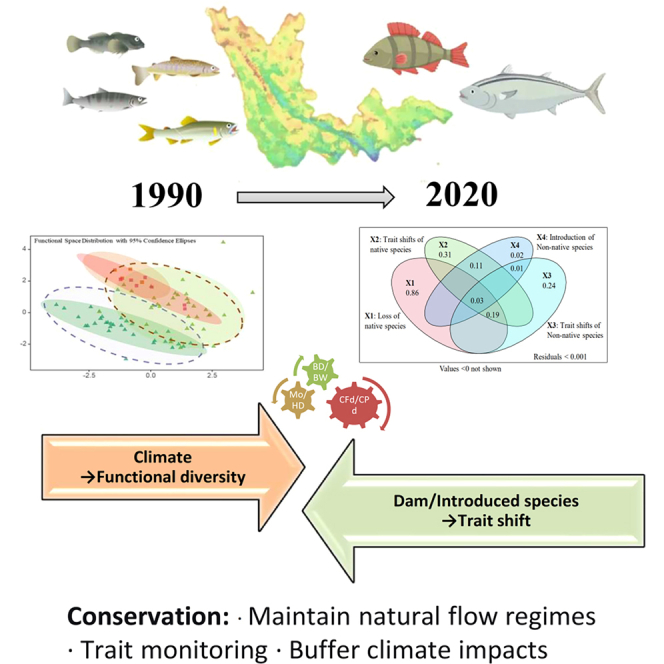

## Introduction

Tropical montane rivers (TMRs) originate in tropical mountains, usually located in Earth’s biodiversity hotspots.^1^ TMRs are characterized by habitat heterogeneity, high biological diversity, endemism, and distinct life-history adaptations of their organisms.^2^^,^^3^^,^^4^^,^^5^ Tropical freshwater ecosystems and their biodiversity are usually affected by anthropogenic activities and environmental changes, including land use change, dam construction, biological overexploitation, climate change, water pollution, and the introduction of non-native species,^3^^,^^6^^,^^7^^,^^8^ which makes them one of the most vulnerable habitats on the planet.^9^^,^^10^ Hence, exploring the environmental characteristics and variabilities of TMRs, while also understanding how they shape the species composition and diversity of current fish assemblages, is a major challenge in conservation biogeography.^11^^,^^12^

Fish assemblages are strongly controlled by historical and geographic factors.^13^ The composition of traits embodies a species’ survival and adaptation strategies in habitat-specific settings.^14^ Its structure was primarily driven by local environmental factors,^15^^,^^16^ which ultimately influence the functional structure of fish communities.^17^^,^^18^ For example, factors such as climate change, water pollution, hydrologic alterations, habitat degradation, and the introduction of non-native species^4^ can cause variations in certain functional traits of fish assemblages, including mobility and swimming performance. These factors can also influence the fish feeding habits and morphology, as well as the phylogenetic relatedness between species within fish assemblages.^19^^,^^20^^,^^21^ Functional diversity provides more specific indications for evaluating community responses to environmental change and making predictions about changes in ecosystem function.^22^^,^^23^

On the other hand, different metrics of functional diversity can capture distinct dimensions of community functional characteristics. For instance, functional richness (FRic) represents the volume of functional space occupied by species in the community, functional evenness (FEve) measures the uniformity of distribution of functional traits within this space, and functional dispersion reflects the degree of dispersion in multidimensional trait distribution.^24^ Significant knowledge gaps remain in understanding the functional structure of tropical freshwater fish assemblages: Key functional traits that respond to environmental changes have not been clearly identified,^18^^,^^25^^,^^26^ and few studies have systematically distinguished the relative contributions of environmental variables versus non-native species introductions to changes in regional fish community composition and functional diversity.

The Yuan-Red River, a tropical mountain river system situated within the Indo-Burma biodiversity hotspot,^1^ has its upper reaches primarily comprising the Yuanjiang, Lixianjiang, and Tengtiaojiang rivers in Yunnan, China. Paleomagnetic evidence suggests potential geological-historical connections between this river system and the drainage networks in southwestern Hainan Island, with its fish fauna composition and variation reflecting the biogeographic evolutionary history of freshwater fishes in the region.^27^ In recent decades, the increasing intensity of water extraction for industrial, agricultural, and domestic uses has led to significant alterations in land use and hydrological conditions throughout the basin.^28^ These changes have consequently triggered a general decline in the distribution and abundance of native species, accompanied by the rapid spread of non-native fish species.^29^ This context underscores the urgency for research on the response of local fish functional traits to these multifaceted pressures.

This study aimed to identify the drivers of changes in fish functional diversity in the Yuan-Red River Basin (YRRB) and to elucidate the roles of environmental conditions and non-native species in shaping the functional diversity of fish assemblages in the basin. We attempt to answer (1) the degree of native fish functional traits shifts and their key driving factors in the YRRB, (2) the contribution of species turnover and the functional trait shifting of native and non-native species on fish functional diversity variation in the YRRB, and (3) the role of environmental conditions and species introduction in explaining changes in species richness (SR) and functional diversity of fish assemblages in the YRRB.

## Results

**Figure 1:**
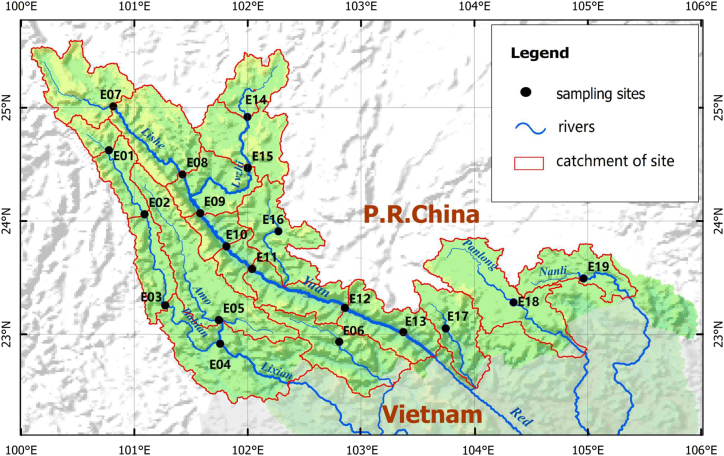
Study area and sampling sites in the Yuan-Red River basin (YRRB)

The Yuan-Red River is an international river that crosses the border between China (Yunnan Province) and Vietnam, with a basin area of approximately 11.3 × 10^4^ km^2^ (Figure 1). Its primary source, the Yuan River, is 677 km long in China, with a natural drop of 2674.6 m and an average riverbed drop of 3.85‰, covering 38073.6 km^2^, accounting for 50.9% of the basin area in China, and 33.7% of the total basin area.^28^ The Yuan River and other tributaries of the Red River (Lixian River, Tengtiao River, Nanxi River, Panlong River, and Pumei/Nanli River) span about three latitude degrees within China, with the elevation dropping from 3,200 m to 76.4 m. The annual rainfall ranges from 700 mm to 3,000 mm. According to historical records, 120 fish species (including four basin endemics) were found in the river system.^30^ Among these, eight species were listed as endangered by the IUCN Red List Categories and Criteria.^31^

A total of 40 species of fish in 38 genera of 15 families and 4 orders were collected in the 2020 survey, including 33 native species in 31 genera of 11 families and 4 orders and 7 non-native species in 7 genera of 5 families and 2 orders. The most abundant families of native species were Cyprinidae (17 species), Bagridae (3 species), Sisoridae (3 species), Balitoridae (2 species), and Nemacheilidae (2 species). The remaining 6 families each had a single species. Among the 7 non-native species, Cyprinidae (3 species) was the most abundant one. The other four species were Channidae, Cichlidae, Odontobutidae, and Xenocyprididae.

### Variation of the traits in history and the current condition

**Figure 2:**
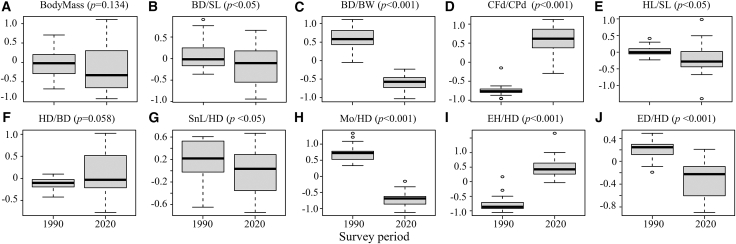
Functional trait variation between the 1990 and 2020 surveys (paired *t* test) (A) Body mass (BodyMass, Wilcoxon single rank test, *p* = 0.134), (B) relative body depth (BD/SL, *p* < 0.05, Cohen’s d = −0.673▲), (C) body transversal shape (BD/BW, *p* < 0.001, Cohen’s d = −4.077 ■), (D) Caudal peduncle throttling (CFd/CPd, *p* < 0.001, Cohen’s d = 4.106 ■), (E) relative head length (HL/SL, *p* < 0.05, Cohen’s d = −0.546▲), (F) relative head depth (HD/BD, *p* = 0.058, Cohen’s d = 0.464♦), (G) relative maxillary length (SnL/HD, *p* < 0.05, Cohen’s d = −0.647▲), (H) oral gape position (Mo/HD, *p* < 0.001, Cohen’s d = −3.616 ■), (I) relative eye position (EH/HD, *p* < 0.001, Cohen’s d = 2.368 ■), and (J) relative eye size (ED/HD, *p* < 0.001, Cohen’s d = −2.143 ■). All traits were log-transformed, and *Z* score standardized to account for allometric scaling. Effect sizes mark: ■ large, ▲ medium, and ♦ small.

According to the *t* test results, eight traits showed significant changes (*p* < 0.05) in the 2020 survey compared to the 1990 survey (Figure 2), including BD/SL, HL/SL, SnL/HD (*p* < 0.05), BD/BW, CFd/CPd, Mo/HD, EH/HD, ED/HD (*p* < 0.001); among them, the mean values of CFd/CPd, EH/HD increased significantly, while the values of BD/SL, BD/BW, HL/SL, SnL/HD, Mo/HD and ED/HD decreased significantly. In addition, the mean values of HD/BD increased (*p* < 0.1).

### Change in functional diversity due to trait variation and species introduction

Within the functional space of ten functional traits, the first two axes of PCoA covered 48.14% of the trait variation (Table S1). The first axis was significantly associated with seven functional traits, including BD/BW (*p* < 0.001), Mo/HD (*p* < 0.001), BD/SL (*p* < 0.001), HD/BD (*p* < 0.001), ED/HD (*p* < 0.01), CFd/CPd (*p* < 0.001), and BodyMass (*p* < 0.001). Meanwhile, the second axis was significantly associated with six traits, including SnL/HD (*p* < 0.001), CFd/CPd (*p* < 0.001), BD/SL (*p* < 0.001), EH/HD (*p* < 0.001), HL/SL (*p* < 0.001), and BodyMass (*p* < 0.05).

**Figure 3:**
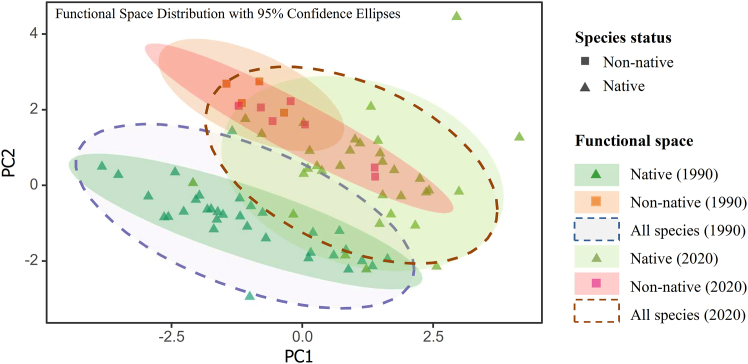
Schematic diagram of changes in the hypervolumes of fish distribution in the Yuanjiang River between the 1990s and 2020s surveys The hypervolumes for different species status are represented using 2D projections, with ellipses indicating the 95% confidence intervals of the functional space. ▲ represent native species, and ■ represent non-native species. The light gray dashed ellipse represents the functional space range of all species in the 1990s survey; the dark green ellipse represents the distribution range of native species in the 1990s; the light orange ellipse represents the distribution range of non-native species in the 1990s. The dark orange unfilled ellipse represents the functional space range of all species in the 2020s survey; the light green ellipse represents the distribution range of native species in the 2020s; the purple-pink ellipse represents the distribution range of non-native species in the 2020s. Overlaps between the 1990 and 2020 surveys: total species pool, 35.2%; native species, 20.6%; non-native species, 24.1%; native and non-native species, 3.7% (1990 survey), 39.5% (2020 survey). See also Table S1.

The hypervolume overlap analysis using the Sørensen overlap index indicated that significant changes were observed in the functional hypervolumes of fish between the 1990 and 2020 surveys in the study area. The functional space hypervolume of the total species pool increased from 154.4 to 320.1 (a 107% growth), with an overlap of 35.2% between the two periods. The functional space of native species expanded from 80.5 to 319.0 (a 296% growth), with an overlap of only 20.6%. The functional space of non-native species increased from 12.0 to 86.6 (a 621% growth), with an overlap of 24.1%. The functional space overlap between non-native and native species was only 3.7% in the 1990s, while it increased to 39.5% in the 2020s. Null model tests indicated that the observed overlap degrees were significantly lower than random expectations (*p* > 0.05), suggesting that the changes in functional space cannot be attributed to sampling bias but rather reflect genuine ecological changes (Figure 3).

**Figure 4:**
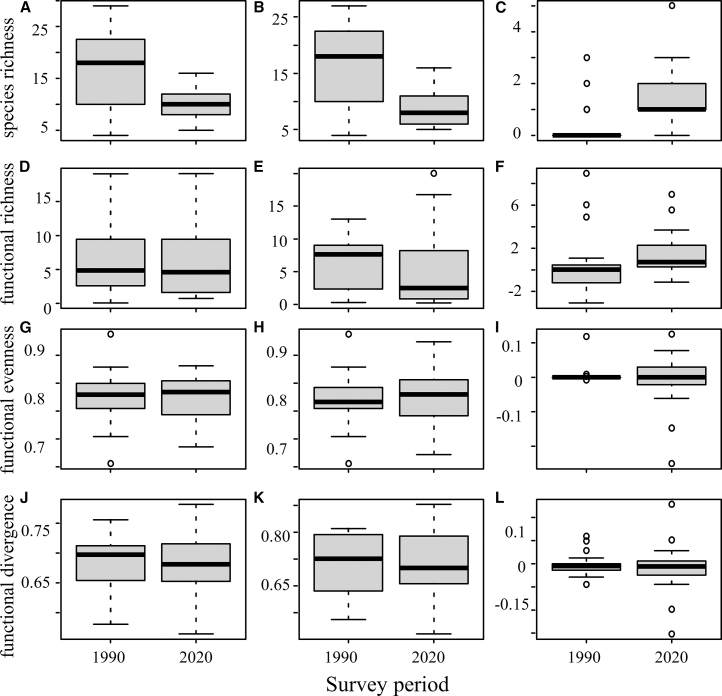
Functional diversity variation between the 1990 and 2020 surveys (paired *t* test) (A) Species richness (SR) of total species (*p* = 0.001, Cohen’s d = −0.893 ■), (B) SR of native species (*p* < 0.001, Cohen’s d = −1.101 ■), (C) SR of non-native species (Wilcoxon signed-rank test, *p* = 0.001), (D) functional richness (FRic) of total species (Wilcoxon signed-rank test, *p* = 0.768), (E) FRic of native species (*p* = 0.524, Cohen’s d = −0.149∗), (F) FRic of non-native species (*p* = 0.331, Cohen’s d = 0.229∗), (G) species evenness (FEve) of total species (*p* = 0.720, Cohen’s d = −0.084∗), (H) FEve of native species (*p* = 0.945, Cohen’s d = 0.016∗), (I) FEve of non-native species (Wilcoxon signed-rank test, *p* = 0.798), (J) functional divergence (FDiv) of total species (*p* = 0.829, Cohen’s d = −0.050∗), (K) FDiv of native species (*p* = 0.755, Cohen’s d = 0.073∗), and (L) FDiv of non-native species (*p* = 0.507, Cohen’s d = −0.155∗). Effect sizes mark: ■ large, ▲ medium, ♦ small, ∗ very small.

**Figure 5:**
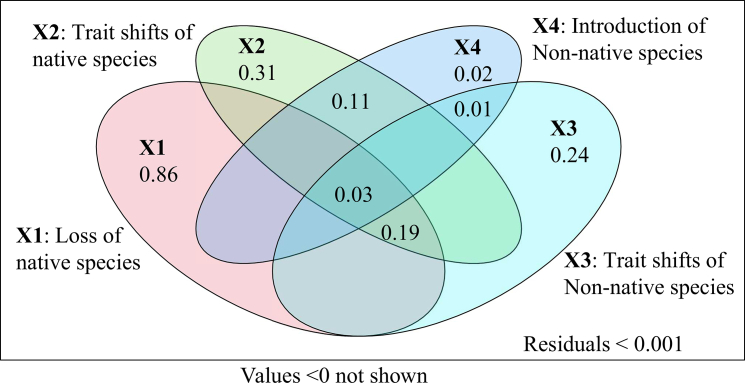
Venn diagram illustrates the results of variation partitioning of fish functional diversity in the YRRB among four components X1, native species loss (adjusted R^2^ = 0.865); X2, traits shifting of native species (R^2^ = 0.309); X3, non-native species introduction (adjusted R^2^ = 0.016); X4, traits shifting of non-native species (adjusted R^2^ = 0.239). Residuals <0.001.

The Paired *t* test results (Figure 4) showed that SR in the study area changed significantly between the 1990 and 2020 surveys. Due to a highly significant decrease in native SR (*p* < 0.001, large effect size), the overall native SR declined markedly, even though non-native SR increased notably. In terms of functional diversity, the overall metrics remained relatively stable: Native species exhibited a slight decrease in FRic (a negative very small effect), while FEve and functional divergence showed minor increases (positive very small effects). For non-native species, FRic increased, and functional divergence decreased (both with very small effects). At the regional scale, overall FRic showed no significant change, whereas FEve and functional divergence decreased slightly (negative very small effects). However, none of these changes in functional diversity metrics were statistically significant. The variation partitioning (VPA) results revealed distinct contributions of four ecological processes to functional diversity changes (Figure 5). All variance inflation factors (VIFs) were <2 (range: 1.41–1.61), confirming minimal collinearity among predictors. Native species turnover explained the largest proportion of variance (adjusted R^2^ = 0.865), followed by shifts in native species traits (adjusted R^2^ = 0.309). Non-native species trait shifts (adjusted R^2^ = 0.239) and turnover (adjusted R^2^ = 0.016) showed smaller contributions. Negative variance components, although present in the full decomposition, were negligible (<0.001) and are interpreted as statistical artifacts arising from complex interdependencies among drivers.^32^

### Environmental factors in explaining the CWM of fish traits shifting

Generalized least squares (GLS) models indicated that the CWM of different fish functional traits in the YRRB were controlled by different environmental factors (Table 1). Traits associated with locomotion (CFd/CPd) were dominated by precipitation (*p* < 0.05) and human population density (POP) (*p* < 0.05), and BD/SL was positively influenced by the number of introduced species (*p* < 0.001). Traits related to feeding and habitat use, including HL/SL, SnL/HD, and EH/HD, were mainly influenced by the number of dams (*p* < 0.05); Among food acquisition traits, Body mass (BodyMass) was negatively affected by dissolved oxygen (DO) content (*p* < 0.01) and the number of dams (*p* < 0.01) but positively associated with the number of introduced species (*p* < 0.001), whereas ED/HD was negatively correlated with chlorophyll-a content (*p* > 0.05). In addition, Mo/HD was primarily influenced by precipitation (*p* < 0.01). The factors that shaped fish functional diversity in the YRRB were unique. SR and FRic were mainly associated with precipitation (*p* < 0.05), FEve was affected by chlorophyll-a content (*p* < 0.01) and mean annual temperature (*p* < 0.05).

**Table 1:** Associations between environmental factors and fish functional traits from generalized least squares (GLS) models

Diversity metrics	Regression coefficients										Model fit		
	Intercept	MAP	MAT	Chl-a	DO	EC	Forest	Dam	POP	Intro	AIC	BIC	logLik
**CWM of functional trait**													

CFd/CPd	**0.605**∗∗∗	**0.162**∗	–	–	–	–	–	–	**0.210**∗	–	22.56	25.65	−7.28
BD/SL	**−0.173**∗	–	–	–	–	0.131	–	–	–	**0.304**∗∗∗	21.66	24.76	−6.83
BD/BW	**−0.590**∗∗∗	–	–	–	–	–	–	–	–	0.095	4.35	6.85	0.83
SnL/HD	−0.034	–	–	–	–	–	–	**−0.167**∗	–	**−0.211**∗∗	19.69	22.78	−5.84
HL/SL	**−0.212**∗∗	–	–	–	–	−0.189	−0.118	**0.478**∗∗∗	–	–	26.84	30.38	−8.42
EH/HD	**0.505**∗∗∗	–	–	–	–	–	–	**−0.205**∗	–	–	23.28	25.78	−8.63
BodyMass^(Spher)^	**−0.186**∗	–	–	–	**−3.08**∗∗	–	–	**−2.62**∗∗	–	**0.516**∗∗∗	33.16	37.41	10.58
Mo/HD^(Gaus)^	**−0.731**∗∗∗	**0.140**∗∗	–	–	–	–	–	–	–	–	6.14	9.48	0.93
ED/HD	**−0.341**∗∗∗	–	–	−0.102	–	–	–	–	–	–	23.54	26.04	−8.76
HD/BD	0.118	–	–	–	–	–	–	0.206	–	–	35.4	37.9	−14.7

**Functional diversity**													

SR	**10.158**∗∗∗	**1.619**∗	–	–	–	–	–	–	–	–	100.13	102.63	−47.07
FRic	**9.869**∗∗	**5.569**∗	–	–	–	–	–	–	–	–	141.14	143.64	−67.57
FEve^(Gaus)^	**0.821**∗∗∗	–	**0.025∗**	**0.03**∗∗	–	–	–	–	–	–	−44.59	−40.73	27.3
FDiv	**0.765**∗∗∗	0.02	–	–	–	–	–	–	–	–	−33.62	−31.12	19.81

Models test the relationship between environmental factors and functional metrics (diversity indices and CWM traits). All models are based on a sample size of *n* = 19. Bold values with asterisks indicate statistical significance: ∗*p* < 0.05, ∗∗*p* < 0.01, and ∗∗∗*p* < 0.001. Responses: CWM of functional trait, Caudal peduncle throttling (CFd/CPd), relative body depth (BD/SL), body transversal shape (BD/BW), relative maxillary length (SnL/HD), relative head length (HL/SL), relative eye position (EH/HD), body mass (BodyMass), relative eye size (ED/HD), relative head depth (HD/BD), and oral gape position (Mo/HD). Functional diversity index, species richness (SR), functional richness (FRic), functional evenness (FEve), functional divergence (FDiv). Predictors: mean annual precipitation (MAP), mean annual temperature (MAT), chlorophyll-a content (Chl-a), dissolved oxygen (DO), electrical conductivity (EC), forest (percent forested land in the catchment), dam (number of reservoirs upstream), human population density (POP), and number of introduced species (intro). All predictor variables were scaled before analysis. Spatial correlation structure: exponential spatial correlation (Exp), spherical spatial correlation (Spher), and Gaussian spatial correlation (Gaus). Model fit: Akaike information criterion (AIC), Bayesian Information Criterion (BIC), and log-likelihood (logLik) indicate the goodness of fit. Residual diagnostics showed no heteroscedasticity and an approximately normal distribution, indicating that spatial structure had been effectively corrected for response variables with spatial auto-correlation. The coefficients in bold were at a significant level of *p* < 0.05. See also Table S4.

## Discussion

As a deep river valley perpendicular to the advancing direction of the humid ocean air mass, the Yuan River Valley developed a Savanna climate with high temperatures and little rainfall, causing the river’s hydrological regime to be highly susceptible to climate change-induced precipitation instability, such as river discharge and suspended sediment.^33^ The towns along the mainstream of the YRRB in China) have experienced a process of increasing POP and deforestation over the past three decades. Although there are no large hydroelectric projects constructed on the mainstream, several small reservoirs were built on the tributaries, especially in the karstic regions in the eastern part of the basin, where surface water is relatively scarce, and cascade hydropower plants were built on the Nanli and Panlong rivers.^28^ Meanwhile, non-native species originally introduced with aquaculture in lakes and reservoirs that emerged after the 1990s have dispersed into natural river channels, resulting in changes to the regional fish fauna in the YRRB.^34^ Dams significantly alter the rivers’ hydrodynamic conditions, water chemistry, and nutrient status, leading to habitat fragmentation and the short-distance migration of some fish species.^29^

### Fish trait variation in relation to environmental factors

Due to their strong morphological plasticity, freshwater fishes provide a robust model for examining phenotypic responses to environmental change.^35^ In the Yuan River Basin (YRRB), precipitation emerged as the dominant factor most strongly associated with fish locomotion traits—specifically CFd/CPd—based on GLS model results (*p* < 0.05). Alongside climatic factors, anthropogenic species introductions are also significantly associated with fish body shape. BD/BW and BD/SL showed a positive correlation with the number of introduced species. Given that introduced fishes are often selected for larger body size,^36^ the observed decrease in these metrics in the YRRB over the past 30 years (with large and medium effect sizes, respectively) highlights a concurrent miniaturization trend in native fish assemblages. This trend coincided with a broader climatic shift toward drier conditions and more frequent droughts since the 2000s.^37^^,^^38^ Declining precipitation is correlated with reduced streamflow and suspended food resources, consistent with global patterns where hydrological variability is correlated with reduced energy intake and smaller body size in fishes^39^—a response also observed in Mediterranean and temperate systems under drought stress.^40^^,^^41^^,^^42^ Furthermore, human POP was positively associated with CFd/CPd (*p* < 0.05), aligning with findings that urbanization can enhance fish swimming efficiency in altered habitats.^43^

Notably, caudal peduncle morphology (CFd/CPd) was associated not only with precipitation but also with forest cover and dam density. Widespread deforestation over the past three decades may have reduced soil water retention, altering runoff patterns and partly buffering the effect of rainfall decline on streamflow.^44^ Concurrently, dam construction has increased water residence time in tributaries, favoring morphological adaptations that enhance sustained swimming.^45^^,^^46^^,^^47^^,^^48^ These overlapping associations suggest that human-modified landscapes can mediate trait-environment relationships in the context of climate—an important consideration for basin-level conservation.

Fish functional traits related to habitat use and feeding showed significant associations with the number of dams based on the GLS model results. Specifically, reservoir environments formed by dams were associated with a shorter relative snout length (SnL/HD, *p* < 0.05) and a ventral shift in eye position (EH/HD, *p* < 0.05). These morphological patterns are consistent with an adaptive shift in predation strategy, potentially from active hunting to more ambush-based or benthic feeding, possibly linked to altered light conditions and foraging perspectives in lentic waters (Haas, 2010). In addition to direct habitat alteration, dam regulation may indirectly relate to trait variation through changes in water chemistry. For instance, regulated flows often increase phytoplankton biomass, elevating chlorophyll *a* and shifting the base of aquatic food webs toward planktonic resources.^49^ While our GLS model did not show a significant direct association between chlorophyll *a* and eye size (ED/HD, *p* > 0.05), body mass was strongly negatively correlated with DO (*p* < 0.01)—a parameter often linked to eutrophic conditions. The pattern of reduced body mass and the trend toward smaller eyes aligns with observations from eutrophic systems in both Europe and China.^50^^,^^51^ These results suggest that eutrophication-related water quality changes, possibly mediated or exacerbated by dams, may impose visual and metabolic constraints that favor smaller-bodied fishes and less visually reliant foraging strategies.

The introduction of non-native species has been further associated with changes in the functional trait structure of fish assemblages. In the YRRB, the prevalence of non-natives is positively correlated with community-wide increases in body mass (BodyMass) and relative body depth (BD/SL) (GLS results, *p* < 0.001). This may reflect the establishment of introduced species with larger body sizes or different ecological strategies, which can alter competitive landscapes and resource availability. Simultaneously, trait shifts related to sensory and feeding structures—such as reduced eye height (EH/HD) and shorter snout length (SnL/HD)—were more strongly associated with the number of dams (*p* < 0.05), highlighting habitat alteration as a primary driver of these specific morphological changes. The combined pressures from non-native species and habitat modification are often linked to the functional homogenization of native assemblages, frequently manifesting as miniaturization and reduced niche breadth, a pattern consistent with observations in invaded river systems worldwide.^52^^,^^53^ Therefore, preserving native functional diversity would benefit from integrated management that addresses both invasive species control and the mitigation of physical habitat alteration.

Unlike many basins where temperature and precipitation are jointly associated with trait variation,^47^^,^^54^^,^^55^ the YRRB’s savanna climate—moderated by foehn effects—suggests precipitation may be a particularly influential climatic factor in this system. Furthermore, locomotion traits showed stronger associations with landscape-scale factors (e.g., precipitation, dams, and POP), whereas feeding traits were more closely linked to habitat-scale water quality parameters. This scale-dependent pattern of association highlights the potential value of multi-level management strategies, consistent with findings from the Pearl River Basin.^55^

### Changes in the functional diversity of fish and their correlates

Over the past three decades, fish assemblages in the Yuanjiang River Basin (YRRB) have exhibited a significant decline in species diversity alongside relative stability in functional diversity (Figure 4), indicating that changes in species numbers did not proportionally alter functional space structure.

This functional stability is strongly linked to the response of the native fish assemblage. Despite a severe loss of native SR (Cohen’s d = −1.101, *p* < 0.001), their FRic did not decrease significantly. The remaining native species showed systematic shifts in key traits—such as decreased body mass and altered mouth position—which were significantly correlated with environmental factors such as dam density and precipitation (Table 1). The substantial expansion of convex-hull volume (+143%) alongside stable functional diversity indices (FRic, FEve, and FDiv) is consistent with a scenario where the functional space expanded in range while maintaining its internal distribution pattern. Native-species dynamics (loss and trait shifts) accounted for 73% of the total functional variation (Figure 5). In contrast, non-native species, while increasing in richness (*p* = 0.001), had a minimal statistical association with FRic expansion (Cohen’s d = 0.229). By 2020, the functional space of non-native species showed a 39.5% overlap with that of native species (Figure 3), suggesting they likely occupied niches released by native species rather than adding new functions. Their spread is facilitated by environmental changes such as flow regulation and warming.

Climate-driven flow alteration has been identified as a key environmental filter associated with functional traits^56^ and is correlated with the success of generalist species, including non-natives.^57^ The loss of native specialists could potentially disrupt long-term interaction networks and reduce ecosystem resilience,^58^^,^^59^ while increased functional overlap with non-natives can diminish community distinctiveness^60^ and promote biotic homogenization.^61^ Thus, the observed functional stability may coincide with a gradual loss of functional redundancy and ecological uniqueness.

### Impact of non-native species on local fish functional diversity

Over 90 native fish species were recorded before 1990 in the YRRB. However, only one-third of them were recorded in the 2020 survey (about 30 species). In addition, more than 25% of the recorded species in the 2020 survey were introduced species. The occurrence of Tilapias*, Channa gachua,* increased significantly. Meanwhile, the occurrence of native species with rare functional traits, such as *Beaufortia leveretti, Pareuchiloglanis macrotrema, Pseudecheneis paviei, Garra orientalis, Gobiobotia yuanjiangensis,* and *Hemibagrus pluriradiatus*, has significantly decreased, especially in the mainstream of the Yuan-Red River.

The data from this study reveal a pronounced shift in the functional patterns of non-native fish in the YRRB between survey periods, characterized by a dramatic expansion of their own functional space (∼600%) and a significant increase in functional overlap with native species (from 3.18% to 40%). This pattern aligns with a dual process whereby non-native species may have contributed to the expansion of the assemblage’s total functional space (+107%) while also exhibiting greater functional similarity to natives. The increase in functional similarity is consistent with the hypothesis of trait convergence under shared environmental pressures, which could potentially contribute to redundancy in certain functional dimensions.^62^ Nevertheless, the 40% overlap and the substantial independent functional volume of non-natives indicate that they retained distinct functional attributes. Taken together, the findings support a composite interpretative framework: non-native species were dynamically involved in the alteration of the community’s functional structure, with their impacts associated with both the expansion of the overall functional space and the adjustment of its internal configuration.

The functional distinctiveness of native fish assemblages has decreased over the past three decades,^63^ a trend consistent with broader patterns of biotic homogenization observed in freshwater systems globally.^64^^,^^65^ In this context, our analysis indicates that traits associated with food acquisition and habitat selection were those most significantly altered in relation to non-native species presence.^53^ While this pattern is consistent with the hypothesis of resource competition between native and non-native species, other drivers, such as shared environmental filtering, cannot be ruled out. Although non-native species were estimated to contribute directly to approximately 16% of the total functional diversity change in the YRRB over 30 years (Figure 5), their role may be amplified by ongoing climate change and cascade hydropower development, which are known to facilitate the colonization and spread of non-native species.^66^^,^^67^ Therefore, even a moderate direct contribution could interact with these major drivers to potentially accelerate reach-scale homogenization,^61^^,^^62^ warranting continued attention in conservation planning.

Our survey indicates that non-native fish have expanded their distribution range within the Yuanjiang River Basin. More importantly, our functional analysis reveals that these widely distributed non-native species have not simply increased redundancy but have significantly altered the functional-structural pattern of the fish assemblage in the basin: They have substantially expanded the overall volume of the functional space while also converging with native species along specific functional dimensions. Given the key role of native species in maintaining the historical functional structure,^68^ the functional reshaping driven by non-native species deserves attention. Future basin management should not only focus on species invasion itself but should also prioritize assessing the functional structural changes it induces and safeguarding native populations with unique functional roles as a primary objective.

### Limitations of the study

(1) The potential effect of gear selectivity on sampling outcomes, along with possible inconsistencies between historical and contemporary survey protocols, may introduce bias in comparisons across time periods, particularly for rare species.
(2) The functional diversity in this study was defined primarily using morphological traits. We acknowledge that a more complete functional characterization should incorporate life-history traits (e.g., reproductive strategies) and physiological traits. However, given the relatively short timescale of this study (30 years), we assumed that community-level functional changes are initially manifested through shifts in species composition and their morphological characteristics, while life-history traits may respond more slowly over longer timescales. Future research integrating multi-dimensional traits is needed to provide a more comprehensive perspective.
(3) This study is based on observational data, revealing strong correlations among species composition, functional traits, and environmental factors. While these patterns align with theoretical expectations—such as climate-driven functional filtering and functional changes resulting from species loss—observational studies alone cannot establish strict causality. Future controlled experiments or comparative studies across watersheds are essential for verifying these potential causal mechanisms.

## Resource availability

### Lead contact

Further information and requests for resources and materials should be directed to and will be fulfilled by the lead contact, Bin Kang (bkangfish@163.com).

### Materials availability

This study did not generate new unique reagents.

### Data and code availability

The datasets and R code supporting this study have been deposited in Figshare under DOI: https://doi.org/10.6084/m9.figshare.31044760. All analyses, including model diagnostics, are fully reproducible using the provided R scripts in the public repository. Any additional information required to reanalyze the data reported in this paper is available from the lead contact upon request.

## Acknowledgments

This work was supported by the 10.13039/501100001809National Science Foundation of China (NSFC) (No. 32460325 and 32060304) and the Yunnan Agricultural Joint Fund Key Project (No. 202301BD070001-133).

## Author contributions

X.X.H.: conceptualization, methodology, writing – original draft, and funding acquisition. A.L.Y.: investigation, data curation, and visualization. B.K.: supervision, conceptualization, and writing – review and editing. K.J.H.: software and validation. X.H.M.: writing – review and editing. W.X.H.: resources and formal analysis. H.D.L.: investigation and validation.

## Declaration of interests

The authors declare no competing interests.

## Declaration of generative AI and AI-assisted technologies in the writing process

During the preparation of this work, the author(s) used DeepSeek to improve code readability and to refine the language of the discussion section. After using this tool, the author(s) reviewed and edited the content as needed and take(s) full responsibility for the content of the publication.

## STAR★Methods

### Key resources table

**Table:** 

REAGENT or RESOURCE	SOURCE	IDENTIFIER
**Deposited data**		

Climatic data	WorldClim (v 2.1)^69^	https://www.worldclim.org
Forest cover data	GlobleLand30	https://globallandcover.com
Population data	WorldPop^70^	https://www.worldpop.org
Dams dataset	GOODD (v 1.0)^71^	https://www.globaldamwatch.org/goodd
Fish catalog	FishBase	https://www.fishbase.org
Fish community and functional trait data	This paper	https://doi.org/10.6084/m9.figshare.31044760
Site-specific environmental data	This paper	https://doi.org/10.6084/m9.figshare.31044760
R code for statistical analyses	This paper	https://doi.org/10.6084/m9.figshare.31044760

**Software and algorithms**		

ArcGIS (v 10.2)	Esri	https://www.esri.com/
R Project for Statistical Computing (v 4.4.2)	The R Foundation	https://www.r-project.org/
car R package (v 3.1–3)	Fox and Weisberg^72^	https://www.john-fox.ca/Companion/
FD R package (v 1.0–12.3)	Laliberté et al.^73^	https://CRAN.R-project.org/package=FD
mFD R package (v 1.0.7)	Magneville et al.^74^	https://doi.org/10.1111/ecog.05904
hypervolume R package (v 3.1.6)	Blonder et al.^75^	https://CRAN.R-project.org/package=hypervolume
vegan R package (v 2.6–8)	Oksanen et al.^76^	https://CRAN.R-project.org/package=vegan
nlme R package (v 3.1–166)	Pinheiro et al.^77^	https://CRAN.R-project.org/package=nlme
MuMIn R package (v 1.48.11)	Bartoń^78^	https://CRAN.R-project.org/package=MuMIn

### Method details

#### Fish data and taxonomic verification

According to the literature, fish surveys conducted across the basin before the 1985–1990 wet season collected small fish using traditional gillnets (mesh size 1–2 cm), while large economic fish were obtained from local fishermen.^79^^,^^80^ The fish surveys from April to June 2020-2021 were also conducted using traditional gill nets (mesh size 1–3 cm). Gill nets were arranged in each site’s upper, middle, and lower reaches. The spatial distribution of the sampling sites is displayed in Figure 1. Large economic fish in the mainstream’s lower reaches were caught with the assistance of local fishermen, ensuring that surveys after 2020 reached at least the same, if not a higher, level of comprehensiveness as those before 1990. The species of historical and current fish samples were identified and validated based on the fish catalog on the FishBase (www.fishbase.org) to avoid misidentification due to invalid species, synonyms, and homonyms. In this study, species that were introduced into natural water bodies via aquaculture (1950s–1960s) and related activities, and have subsequently established and spread, are considered non-native species.

#### Functional traits measurements

To reveal the impact of environmental changes on the functional diversity of fishes, we measured morphometric data of fishes, including body weight (body mass), body standard length (SL), body depth (BD), body width (BW), head length (HL), head depth (HD), EH, eye diameter (ED), snout length (Snl), mouth opening (Mo), caudal fin depth (CFd), caudal peduncle depth (CPd). The mean value of the measured trait metrics was used to calculate the value of each functional trait for the species in this survey. The morphometric data for the 1990 survey were obtained from morphometric measurements of historical fish specimens, while those for the 2020 survey were collected through recent field sampling. For each studied species, at least 30 sexually mature fish samples were measured. However, measurements were taken of all available samples for the species with fewer than 30 samples. A total of 10 morphological traits were chosen for functional diversity analysis, reflecting the functional differences in habitat use, locomotion, swimming abilities, and food resource utilization among the fish groups (Table S2). All traits were log-transformed and *Z* score standardized to ensure equal weighting in the construction of the functional space.

#### Environmental data collection

A total of nine environmental variables from five categories were chosen, including climatic conditions (mean annual temperature and annual precipitation), water chemical parameters (dissolved oxygen, chlorophyll-a, electrical conductivity), landscape-scale variables (percent of forest land in the catchment), and anthropogenic factors (population density in catchment, number of dams in upstream catchment), and biological invasion parameter (species numbers of introduced fish) (Table S3). The climatic data, including annual mean temperature (°C) and mean annual precipitation (mm), were directly extracted from the *WorldClim* database (version 2.1)^69^ at a 1-km spatial resolution for the geographic coordinates corresponding to each sampling site. The forest cover data derived from 2020 land cover data at 30 m resolution was obtained from *GlobleLand30* (globallandcover.com), and 2020 population data at 1 km resolution were obtained from *WorldPop* (www.worldpop.org).^70^ The dams dataset with geospatial coordinates was obtained from the *Global Geo-referenced Database of Dams* (*GOODD Ver 1.0*).^71^ All the data were spatially extracted based on the upstream catchment area of each sampling site. Water chemical data for the study sites were obtained from field surveys. Fieldwork was undertaken during the dry season of April 2020, which had stable flow conditions. The water quality sampling sites were set at the exact locations of the fish survey (Figure 1). At each site, the water electrical conductivity (EC, μS·cm^−1^) and dissolved oxygen (DO, mg·L^−1^) were measured on-site using a portable YSI probe (YSI 6600). Then, the water samples were transported to the laboratory at 0 °C, and the chlorophyll *a* content (Chl-a, μg/L) was analyzed using a visible spectrophotometer (HJ 897–2017) immediately upon arrival at the laboratory. The biological invasion parameter was expressed as the number of introduced species at the sampling sites, where “0” denoted the absence of such species.

### Quantification and statistical analysis

#### Paired t-tests for the difference

Differences in morphological traits of native fish species between the two survey periods (1990 vs. 2020) across the 19 sampling sites were assessed using paired comparisons. Statistical significance was set at *p* < 0.05. Following preliminary checks for normality (Shapiro-Wilk test) and homogeneity of variances (Levene’s test), paired t-tests were applied to the data, which met all parametric assumptions; otherwise, the Wilcoxon signed-rank test was employed as a distribution-free alternative. To quantify the magnitude of observed shifts, effect sizes (Cohen’s d) were calculated for traits showing significant changes over the 30-year period. These analyses, along with comparisons of group means for identified traits, were performed in the R software (version 4.4.2, www.R-project.org) using the *car* package.^72^

#### Functional trait-based indices calculation

Community-weighted means (CWM) reflects the dominant trait value and is often used to quantify shifts in such values along different environmental conditions.^81^^,^^82^^,^^83^ The CWM of each sampling site was calculated as follows^84^:

$$\text{CWM}=\sum_{i=1}^{s}P_{i}\times X_{i}$$

Where *Pi* represents the relative abundance of species *i* in the fish assemblage (one sample site), and *Xi* represents one functional trait of fish species *i* in one particular sample site.

Functional diversity refers to the distribution of species within the functional space of a community, as well as their relative abundance within that space. Functional richness (Fric) is the functional space occupied by a community of species, calculated as the convex hull volume encompassing all species in the multidimensional trait space.^85^ Functional evenness (FEve) quantifies the regularity of abundance distribution within the functional space, with lower values indicating uneven resource use.^86^ Functional divergence (FDiv) measures how abundance is distributed within the functional space, with higher values indicating that abundant species occupy extreme trait values.^86^

Community-weighted mean (CWM) trait values and three functional diversity indices (FRic, FEve, FDiv) were calculated using the *FD* package^73^ and validated with the *mFD* package.^74^ All calculations were based on the standardized trait matrix and species abundance data for each site and survey period.

#### Variance partitioning of functional diversity change

Changes in the species composition of a community over time, as well as shifts in functional traits, can lead to overall changes in the volume of the community in functional space, i.e., changes in community functional richness.^18^ If the species composition of the fish assemblage does not change, the variation in the functional convex hull volume suggests a shift in traits among these fish.

In the functional space constructed by the ten functional traits, we calculated the volume of the whole functional convex hull occupied by fish species for the two surveys. We measured the following four pairs of shifts between two convex-hull spaces: native species in the 1990 survey and the 2020 survey; non-native species in the 1990 survey and the 2020 survey; native and non-native species in the 1990 survey; native and non-native species in the 2020 survey. The functional richness shift was partitioned into four components (X1, X2, X3, X4) based on different causes. The X1 component represents native species turnover. The X2 component represents the shifting of native species' traits. The X3 component represents the turnover of non-native species. The X4 represents the trait shifts of non-native species.

When calculating functional richness, keeping the species composition of the community unchanged while altering the functional trait matrices of the species resulted in the component of variation in functional diversity due to functional trait shifts (X2 and X4); meanwhile, when the species composition matrix was altered keeping the functional trait matrices unchanged, the component of variation in functional diversity related to species turnover was obtained (X1 and X3).

Additionally, the species turnover and trait shifts of both native and non-native species affect functional diversity variation. Variation partitioning (VPA) was used to identify the contributions of native and non-native species. A schematic diagram that summarizes the key steps involved in VPA is shown in Figure S1. The hypervolumes calculation without dimensionality reduction using was conducted using the *hypervolume* package in the R software.^75^ Null models (999 permutations) tested sampling bias significance, and bootstrap resampling (999 replicates) quantified uncertainty. The VPA calculation was conducted using the *vegan* package^76^ in the R software.

#### GLS: Traits and functional diversity drivers

To account for potential spatial autocorrelation in our data, we employed generalized least squares (GLS) models, which are well-suited for such analysis.^87^ Prior to model fitting, we tested the residuals of preliminary linear models for both functional traits and diversity indices for spatial autocorrelation using Moran’s I (Table S4). For variables exhibiting significant spatial autocorrelation, we compared GLS models incorporating exponential, Gaussian, and spherical spatial correlation structures. The spatial structure yielding the model with the lowest Akaike Information Criterion (AIC) was selected for subsequent analysis. Variables without significant spatial autocorrelation were fitted using a GLS model without a spatial structure. To avoid multicollinearity, we calculated the VIF for all candidate environmental predictors. Only variable combinations with VIF <5 were retained for model construction.^88^ All possible models were then generated and ranked by AIC using a dredging procedure. To ensure model robustness and prevent overfitting, we applied strict criteria: we performed model averaging on all models with ΔAIC <2, retained only predictors with a relative importance (sum of Akaike weights) > 0.5, and constrained the maximum number of parameters in the final model based on sample size.^89^ The optimal model was ultimately selected based on a comprehensive evaluation of AIC, Bayesian Information Criterion (BIC), residual diagnostics, and ecological interpretability. All statistical analyses were conducted in R software (version 4.4.2). GLS models were fitted using the *nlme* package,^77^ and model dredging and averaging were performed using the *MuMIn* package.^78^ All models are based on a sample size of *n* = 19. Statistical significance was set at *p* < 0.05, *p* < 0.01, and *p* < 0.001.
