# Supplementary material for: Functional diversity of fish in the Yuan-Red River: Role of species turnover and trait shifts
Source: iScience. 2026 Mar 20;29(4):115418. doi: 10.1016/j.isci.2026.115418 (PMC13087787; doi:10.1016/j.isci.2026.115418)
Supplement: Document S1. Figure S1 and Tables S1–S4 [file mmc1.pdf]

iScience, Volume *29*

Supplemental information

Functional diversity of fish in the Yuan-Red River: Role of species turnover and trait shifts

Xiao-Xia Huang, Ai-Ling Yang, Bin Kang, Ke-Jian He, Xiao-Han Mei, Wen-Xian Hu, and Hung-Du Lin

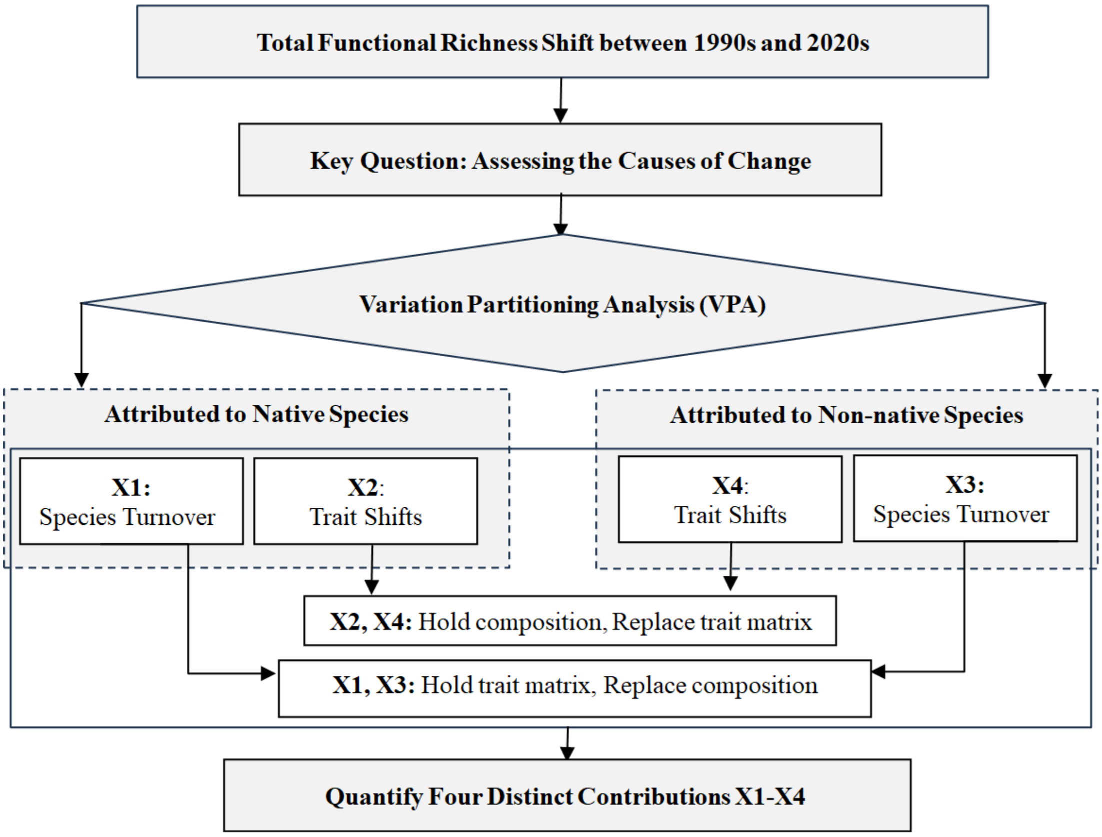

Figure S1 Framework for attributing functional diversity changes to native and non-native processes via Variation Partitioning Analysis (VPA). The total observed change in functional diversity (ΔFD) between periods is conceptually decomposed into four independent contributions: native species turnover (X1), native trait shifts (X2), non-native species introduction (X3), and trait differences of non-natives (X4). Each component is quantified by calculating differences in functional diversity indices (e.g., functional richness) under specific combinations of period-specific species composition and trait matrices. The complete computational process and code for deriving X1–X4 are provided in the **RESOURCE AVAILABILITY** section. **Related to STAR Methods.**

Table S1 Relations between trait and PCoA axes.

trait	axis	R^2	p.value
BD.BW	PC1	0.704	<0.001
Mo.HD	PC1	0.506	<0.001
BD.SL	PC1	0.408	<0.001
HD.BD	PC1	0.395	<0.001
ED.HD	PC1	0.262	<0.001
CFd.CPd	PC1	0.225	<0.001
BodyMass	PC1	0.184	<0.001
Snl.HD	PC2	0.677	<0.001
CFd.CPd	PC2	0.376	<0.001
BD.SL	PC2	0.336	<0.001
EH.HD	PC2	0.235	<0.001
HL.SL	PC2	0.217	<0.001
BodyMass	PC2	0.158	<0.001
HD.BD	PC3	0.519	<0.001
HL.SL	PC3	0.507	<0.001
EH.HD	PC3	0.439	<0.001
BodyMass	PC3	0.119	0.0022
CFd.CPd	PC3	0.094	0.0067
EH.HD	PC3	0.439	0.0432

The cumulative percentage of variance explained by the first two PCoA axes was 48.14%, first three PCoA axes were 66.24%. The correlations between traits and PCoA axes were tested using linear models, and five traits showed significant relationships with the first three PCoA axes, **Related to Figure 3.**

Table S2 Functional trait formulas and description of functional role, Related to STAR Methods.

Functional trait	Functional group	Formula (Code)	Functional role
Relative body depth	locomotion, habitat use	BD/SL	Reflects the physiological ability to deal with mobility, food acquisition, defense against predation, and habitat location [S1],[S2].
Body transversal shape	locomotion, habitat use	BD/BW	A proxy of vertical position in the water column, most benthic fishes are vertically flattened or rounded, while most benthopelagic fishes have deep bodies [S2],[S3].
Caudal peduncle throttling	locomotion, habitat use	CFd/CPd	Caudal propulsion efficiency through reduction of drag [S1],[S2].
Relative head length	habitat use, food acquisition	HL/SL	High values may indicate fish able to feed on relatively larger prey [S4].
Relative eye position	habitat use, food acquisition	EH/HD	Reflects the position of fish and/or its prey in the water column [S1],[S5].
Relative maxillary length	food acquisition	SnL/HD	Size of mouth and strength of jaw[S1],[S6].
Oral gape position	food acquisition	Mo/HD	Position of prey in the water [S3],[S7].
Relative head depth	food acquisition	HD/BD	Hydrodynamism and head size [S1],[S6].
Relative eye size	food acquisition	ED/HD	Visual acuity, relating to prey detection [S1].
Body mass	habitat use, locomotion food acquisition	BodyMass	Reflects the size and metabolism [S5],[S7].

Table S3 Habitat and landscape scale environment variables recorded for the Yuan-Red River survey site, Related to STAR Methods.

Category	Abbreviation	Variable	unit
Climate variables	MAT	Mean annual temperature in the catchment	℃
Climate variables	MAP	Annual precipitation in the catchment	mm
Water chemical	Chl-a	Content of chlorophyll-a	$\mu g \cdot L^{-1}$
Water chemical	EC	Electrical Conductivity	$\mu S \cdot cm^{-1}$
Water chemical	DO	Instantaneous dissolved oxygen	$mg \cdot L^{-1}$
Landscape variables	Forest	Percentage of forested land in the catchment	%
Anthropogenic variables	POP	Mean population density in the catchment	$person \cdot km^{-2}$
Anthropogenic variables	Dam	Number of dams in upstream catchment	$dams \cdot km^{-2}$
Biological invasion	Intro	Number of non-native species at a site	count

Table S4 Spatial autocorrelation of functional traits result (Moran'I test).

Index	Moran_I	Expected_I	Variance	p_value
BodyMass	0.363	-0.056	0.016	0.001
BD.SL	0.054	-0.056	0.017	0.197
BD.BW	0.148	-0.056	0.016	0.055
CFd.CPd	-0.197	-0.056	0.015	0.875
HL.SL	0.077	-0.056	0.014	0.132
HD.BD	-0.055	-0.056	0.017	0.499
Snl.HD	0.052	-0.056	0.017	0.204
Mo.HD	0.231	-0.056	0.015	0.010
EH.HD	0.047	-0.056	0.013	0.187
ED.HD	-0.016	-0.056	0.017	0.379
SR	-0.043	-0.056	0.017	0.460
FRic	0.075	-0.056	0.014	0.134
FEve	0.186	-0.056	0.016	0.029
FDiv	-0.289	-0.056	0.016	0.968
FDis	0.007	-0.056	0.015	0.305

Moran's I test results showed that among the 18 trait indices, three indices (BodyMass, Mo/HD, FEve) exhibited significant spatial autocorrelation ($p < 0.05$), while the remaining 12 indices showed no significant spatial autocorrelation. Based on these results, we applied a spatially structured GLS model to variables with significant spatial autocorrelation, **Related to Table 1**.

Supplemental References

[S1]Brosse, S., Charpin, N., Su, G., Toussaint, A., Herrera-R, G.A., Tedesco, P.A., and Villeger, S. (2021). FISHMORPH: A global database on morphological traits of freshwater fishes. Global Ecology and Biogeography *30*, 2330–2336. https://doi.org/10.1111/geb.13395.

[S2]Villéger, S., Brosse, S., Mouchet, M., Mouillot, D., and Vanni, M.J. (2017). Functional ecology of fish: current approaches and future challenges. Aquat Sci *79*, 783–801. https://doi.org/10.1007/s00027-017-0546-z.

[S3]Sibbing, F.A., and Nagelkerke, L.A.J. (2000). Resource partioning by Lake Tana barbs predicted from fish morphometrics and prey characteristics. Reviews in Fish Biology and Fisheries *10*, 393–437. https://doi.org/10.1023/A:1012270422092.

[S4]Watson, D.J., and Ballon, E.K. (1984). Ecomorphological analysis of fish taxocenes in rainforest streams of northern Borneo. Journal of Fish Biology *25*, 371–384. https://doi.org/10.1111/j.1095-8649.1984.tb04885.x.

[S5]Qiao, J., Liu, Y., Fu, H., Chu, L., and Yan, Y. (2022). Urbanization affects the taxonomic and functional alpha and beta diversity of fish assemblages in streams of subtropical China. Ecological Indicators *144*, 109441. https://doi.org/10.1016/j.ecolind.2022.109441.

[S6]Toussaint, A., Charpin, N., Brosse, S., and Villéger, S. (2016). Global functional diversity of freshwater fish is concentrated in the Neotropics while functional vulnerability is widespread. Sci Rep *6*, 22125. https://doi.org/10.1038/srep22125.

[S7]Shuai, F., Lek, S., Li, X., and Zhao, T. (2018). Biological invasions undermine the functional diversity of fish community in a large subtropical river. Biological Invasions 20, 2981–2996. https://doi.org/10.1007/s10530-018-1751-y.
